# Mitigating Cold Start Problem in Serverless Computing with Function Fusion

**DOI:** 10.3390/s21248416

**Published:** 2021-12-16

**Authors:** Seungjun Lee, Daegun Yoon, Sangho Yeo, Sangyoon Oh

**Affiliations:** Department of Artificial Intelligence, Ajou University, Suwon 16499, Korea; henry174@ajou.ac.kr (S.L.); kljp@ajou.ac.kr (D.Y.); soboru963@ajou.ac.kr (S.Y.)

**Keywords:** serverless computing, function fusion, serverless workflow

## Abstract

As Artificial Intelligence (AI) is becoming ubiquitous in many applications, serverless computing is also emerging as a building block for developing cloud-based AI services. Serverless computing has received much interest because of its simplicity, scalability, and resource efficiency. However, due to the trade-off with resource efficiency, serverless computing suffers from the cold start problem, that is, a latency between a request arrival and function execution. The cold start problem significantly influences the overall response time of workflow that consists of functions because the cold start may occur in every function within the workflow. Function fusion can be one of the solutions to mitigate the cold start latency of a workflow. If two functions are fused into a single function, the cold start of the second function is removed; however, if parallel functions are fused, the workflow response time can be increased because the parallel functions run sequentially even if the cold start latency is reduced. This study presents an approach to mitigate the cold start latency of a workflow using function fusion while considering a parallel run. First, we identify three latencies that affect response time, present a workflow response time model considering the latency, and efficiently find a fusion solution that can optimize the response time on the cold start. Our method shows a response time of 28–86% of the response time of the original workflow in five workflows.

## 1. Introduction

Artificial Intelligence (AI) has become a vital part of our everyday life. Long before AlphaGo [1] surprised us by beating the top class Go player Se-dol Lee in 2016, AI had already been applied to help us to improve search results [2], enhance recommendation quality of online shopping [3], and build human-like robots [4], among other things. Although machine learning and deep learning are recognized as the key technologies for the current advancements made in AI, other technologies such as big data and high-performance computing could also play a crucial role.

With AI set to become a “must-be-included” technology in every application, we need AI computing infrastructure that provides environments for training models, managing data, offering an inferencing interface, etc. MLOps, extended from DevOps, is a recent trend that includes practices of deploying and managing machine learning models. MLOps aims to provide machine learning models in production level in automated and reliable manner [5]. Similar to DevOps, enabling MLOps requires many tools and platforms that are operated in a cloud environment. Serverless computing is yet another cloud-based software platform architecture that enhances AI services.

Serverless computing is a novel cloud-based code-execution paradigm that is executed in an on-demand fashion by a cloud provider. Because of its flexible use of computing resources and auto-scaling capability, it is well suited for AI services (mostly for inferencing) as well as edge computing applications that utilize AI (for inferencing, collecting data, off-loading intensive computations, etc.) In serverless computing, a function does not occupy any resource when it is idle, and the required resource will be allocated instantly by the cloud provider. After the execution, the resource will be reclaimed. In contrast, during times of high demand (i.e., many request to the function), the serverless platform replicates the function object and runs them concurrently. Thus, applications can be run in a cost-effective (i.e., charging based on the time of running), flexible, and scalable (i.e., auto-scaling) manner on serverless platforms.

However, there are downsides too. The first one is execution time limits. If serverless computing is used on a public cloud instead of on-premises platform, a function has a maximum execution time limit. It is because the cloud provider would provide a transient resource such as spot instances in Amazon Web Service (AWS) for serverless functions. The second is a cold start problem [6,7], a trade-off with on-demand resource usage pattern. The cold start problem means an overhead latency to provision when a function is requested for the first time. As mentioned above, an idle function does not reserve any resource, and resource provisioning starts when the first request arrives. Hence, there is a latency between request arrival and actual function execution initiation. This latency is called cold start latency.

**Figure 1:**
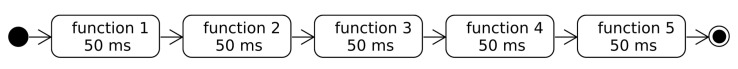
A function chain with five 50 ms functions. If the cold start latency is assumed as 100 ms, the response time is 750 ms for the first time.

As AI services or applications, especially for edge computing, do not consist of a single function but rather a workflow, the cold start problem can cause serious performance degradations. The cold start latency of each function call from a workflow will be accumulated along the function chain. This accumulated cold start latency becomes severe when the function chain is long. For example, suppose there are five functions in the workflow in a linear chain. The function execution time is 50 ms, the cold start latency is 100 ms in this example. As shown in Figure 1, when the first request triggers this workflow, the response time is 750 ms as there are five cold start latencies. The response time of the second request to the chain will be reduced to 250 ms because there is no cold start from now on. According to Daw et al., 2020 [8], the cold start latency can account for 46% of the workflow response time if the workflow consists of 5 s functions and up to 90% of the workflow response time if the workflow consists of 500 ms functions.

As the cold start latency is a critical problem that hinders the wider use of application platforms, many studies have been conducted to address it. One early but notable study focused on reducing the cold start latency of a workflow is Daw et al. [8], wherein the authors proposed a speculative function preloading method called Xanadu. In the proposed method, a preloading schedule (i.e., when and what function should be loaded) is calculated using the profile data with a function execution time, warm start latency, and branch probability. The experiment results of the study showed that Xanadu could reduce the cold start latency by 20% compared with Knative, while minimizing resource over-provisioning caused by preloading simultaneously. However, to apply Xanadu, a modification should be made to a serverless platform and re-deployed. Hence, a user who uses a commercial serverless platform (e.g., Lambda of AWS [9]) cannot take full advantage of Xanadu. Function fusion [10] is another proposed scheme that can be applied to serverless services/applications on the public cloud. Function fusion is one of the function composition methods, and it fuses two or more functions into a single fused function. Fusion can reduce cold start latency, as there is only one cold start latency for loading a fused function (i.e., a similar effect to preloading functions). One of the most popular function fusion methods is Costless [11], in which Elgamal et al. proposed a method that can exploit memory configuration and IoT devices for fusing functions. However, it can handle only a sequential workflow, which implies that it cannot consider a branch in a workflow and parallel or concurrent execution of a function.

**Figure 2:**
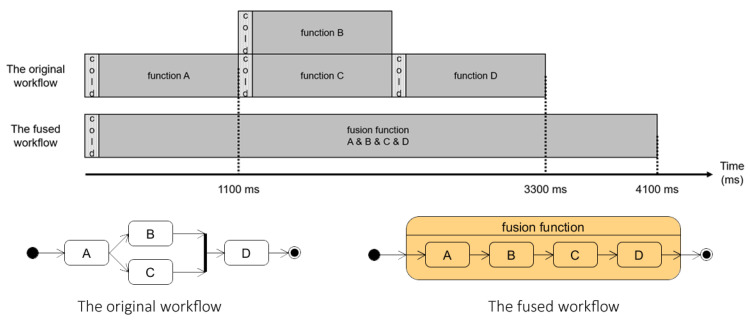
Workflow in this figure has two functions that can be run in parallel. Let the cold start latency be 100 ms and the execution time of functions be 1000 ms. In this case, if all functions in the workflow are fused into a single function, the workflow response time is increased because the functions B and C have to be run in sequence.

Fusing a workflow with a possible parallel or concurrent execution of functions must be addressed carefully, as function fusion may increase the execution time, which, in turn, may result in significant performance degradation. For example, suppose that there is a workflow with four functions shown in Figure 2. The two functions in the middle of the workflow can be executed concurrently, the execution time of each function 1000 ms and the cold start latency is 100 ms. Without a fusion, the response time is 3300 ms (cold start latency of 300 ms + an execution time of 3000 ms), and the response time will be increased to 4100 ms after fusion (cold start latency 100 ms + execution time 4000 ms). Although the cold start latency is reduced, the overall performance is decreased due to the loss of parallelism.

To address the branching and parallel execution issues of the workflow execution in serverless environment, we propose a scheme that reduces the cold start latency of a workflow that contains a branch and a possible parallelism with function fusion technique. The main contributions of this study are summarized as follows:

- We propose a model of the workflow response time in the cold start and the warm start modes as well as in sequential and parallel runs (Section 3).
- We propose a function fusion scheme that handles branch and parallel execution fusing (Section 4).
- We present a practical fusion automation process for stateless functions. Even when implemented for AWS Lambda, the proposed fusion automation process can be easily adapted for other serverless platforms (Section 4).
- We evaluated the performance of the proposed method by conducting thorough evaluations on real cloud environment (i.e., AWS Lambda).

To the best of our knowledge, this study is the first attempt to reduce workflow cold start latency through function fusion.

The rest of the paper is organized as follows. We present the background of the topic covered in this study in Section 2. In Section 3, we present the formulation of the problem and modeling. The proposed function fusion scheme is described in Section 4. In Section 5, experiments and analysis of the results are presented. In Section 6, some related works are explained. Finally, in Section 7, we present our conclusions and directions for future research.

## 2. Background and Motivation

### 2.1. Composing a Workflow Using Function Fusion

A function, a procedure, or a method in serverless computing is a module that contains a set of instructions to achieve a task. These independent functions form a workflow for more complex tasks. A workflow can be formed using existing functions or by adding functions from a library. With this code reusability as well as task-oriented coding features (i.e., not worrying about systematic issues such as fault tolerance, load balancing, handling HTTP/S requests), a service or an application in serverless environment can be developed with high productivity. There are several methods for functions to work together. Baldini et al., 2017 [10] identified three methods: composition by reflective invocation, composition by fusion, and composition by triggering. Reflective invocation uses API provided by the serverless platform to invoke sub-functions. In the triggering method, a function emits an event to invoke a sub-function. The difference between the two methods is that reflective invocation is synchronous and triggering is asynchronous.

Function fusion is a function composition method that fuses two or more functions into a single function. Fusion has advantages compared with reflection and triggering. As noted in the Introduction section, we can reduce cold start latency by using function fusion because loading a fused function shows a similar effect to function preloading. Furthermore, there is no need to use a scheduler function that is used in the reflective invocation method, where a scheduler function is required to handle requests and responses of sub-functions. The scheduler function runs concurrently with sub-functions. Thus, one has to pay for executions of the scheduler function as well. This duplicate charging is called the double-billing problem [10]. Another advantage of using function fusion is that there is no need to modify the code. In the triggering method, a function should be modified to emit an appropriate event to make a chain by event triggering. However, it is not necessary in the case of function fusion, as source functions are not touched (i.e., altered) to build a fused function.

Although fusion has some advantages over other methods, there are limitations to using function fusion. As noted, there is a limitation on the maximum execution time in serverless platforms of public clouds. Thus, if the total sum of the execution time of source functions exceeds the maximum execution time, the functions should not be fused. However, we do not have to consider the maximum memory limitation because each source function satisfies the maximum memory limitation; as these functions are executed in a sequence in the fused function, the fused function will not exceed the maximum memory limitation. Someone may point that a function fusion increases the cost of execution as it requires more memory. When two functions with different memory requirements are fused, the memory requirements of the fused function must be set to match the demands of the larger of the two functions. This causes an increase in the cost. Although this is a concern, the cost factor in function fusion is outside the scope of this study because the cost model is highly dependent on commercial serverless platforms. In this study, we instead focus on correct and efficient execution of a given workflow.

### 2.2. Cold Start and Warm Start

The cold start problem in serverless computing is the main obstacle we tackle in this study. Suppose that a user uploads a new function and the first request to the function arrived after some time. As the serverless platform does not allocate any resource to idle functions, it will allocate required resources upon the request and deploy a new function instance to handle the request. Thus, there is an unavoidable delay between the request arrival and the function execution. This problem in the function invocation is called *cold start*. In contrast, the serverless platform can handle requests instantly from the second request as far as the platform holds resources for the function instance. This type of invocations is called *warm start*.

In some definitions, cold start latency refers to the execution delay due to launching a new function instance because there are no ready resources [12,13]. Alternatively, in some other definitions, cold start latency includes other delays such as resource provisioning, networking delays, user-space setup, and orchestration delays [8]. In this study, we follow the first definition of cold start latency. That is, the cold start latency is the time difference between the execution delay on the cold start and the execution delay on the warm start.

The degree of cold start (i.e., how severe it is) is affected by the provisioning environment, startup process, code language, allocated memory size, and code size [8,14]. If a user is not allowed to configure the serverless platform (e.g., a commercial serverless), environment provisioning and process startup are not in the user’s hands. Changing the code is the main way to change the degree of cold start [14]. However, it is hard to change the code language in practice if the language has already been selected. If we allocate more memory to the function, we will have shorter cold start latency in the linear fashion (i.e., more memory will make latency shorter). However, more memory allocation leads to an increase in cost, and thus, it is not a feasible solution. The code size of a function can affect its cold start latency. Surprisingly, a large code shows a shorter latency. However, the reduction in latency is merely noticeable. For example, a 185 KB function shows a 10.56 ms cold start latency, and a 14.3 MB function shows a cold start latency of 6.47 ms. If the code size is increased by 80 times, the cold start latency is decreased by only 4 ms; therefore, the amount of cold start latency that can be practically reduced by increasing the code size is not significant. This is why the cold start problem is more severe for a function with a short execution time. According to Du et al. (2020), cold start latency can take up to 70% of a function’s lifetime [15]. For these reasons, we consider cold start latency as a constant value regardless of functions.

A cold start can still occur for the second request. Serverless platforms reclaim resources of idle-state functions (i.e., functions that do not receive any request during some period) to increase resource utilization. In AWS Lambda, it is 10 min, and it is 20 min for Microsoft’s Azure Function [8]. If a request arrives slower than this time frame, the requested function experiences a cold start for every request. Further, even if a function is requested frequently enough, a cold start can still occur because of auto-scaling. When the request rate increases, a serverless platform automatically deploys a new copy of the requested function instance. As this will be the first request from the perspective of the copy, it will result in a cold start.

## 3. Problem Modeling

In this section, we describe our problem modeling and the several elements of our proposed algorithm. For the cold start mitigating scheme, we define a model to calculate the cold start latency of a given workflow and design an algorithm to find near-optimal fusion decisions to fuse for a workflow. All symbols used in this section are summarized in Table 1.

### 3.1. Graph Representation of a Workflow

**Figure 3:**
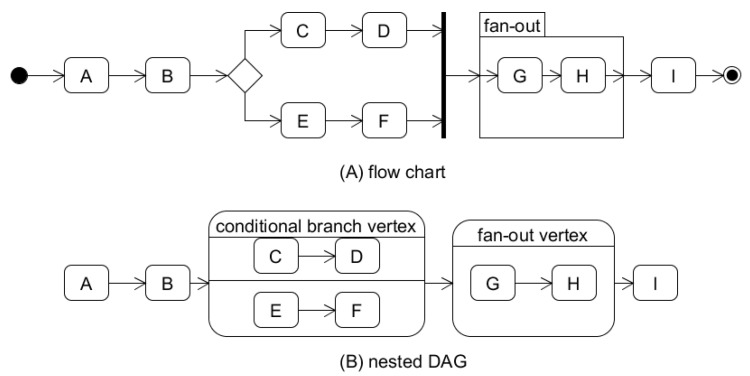
Example to show the nested and directed acyclic graph representation. A fan-out or a conditional branch has its sub-workflow, and its sub-workflow is inside of the vertex representing it. Hence, the overall workflow graph is linear, has only one source vertex, and one sink vertex.

We model the workflow as a nested directed acyclic graph where the vertex represents a workflow item, and the directed edge shows call dependency. Some workflow items (fan-outs and conditional branches, explained below) have their own sub-workflow in our workflow model. Hence, the workflow can appear nested, and accordingly, the workflow graph is also represented in a nested shape. As each item has only one entry point and one endpoint, the overall appearance of the graph is represented linearly. In the case of fan-outs and the conditional branches, there is another linear graph inside the vertex. Figure 3 shows a nested DAG representation of a workflow.

Our technique targets workflows in the form of a pipeline so that fusing can be applied to all functions of the workflow. A workflow should satisfy the following constraints:

- There can be only a single starting point as well as a single endpoint in the workflow.
- The split execution of the workflow that will end at different points is not allowed. In addition, two execution flows that start from different points and meet in the middle are not allowed either. Hence there is one source vertex and one sink vertex in a graph.
- Reversing the workflow is not allowed.
- Only parallel execution of the same structure (fan-out) is possible. If parallel execution of different structures is required, fan-out with a conditional branch should be used.
- Each branch of a conditional branch should be joined at the same point.

**Figure 4:**
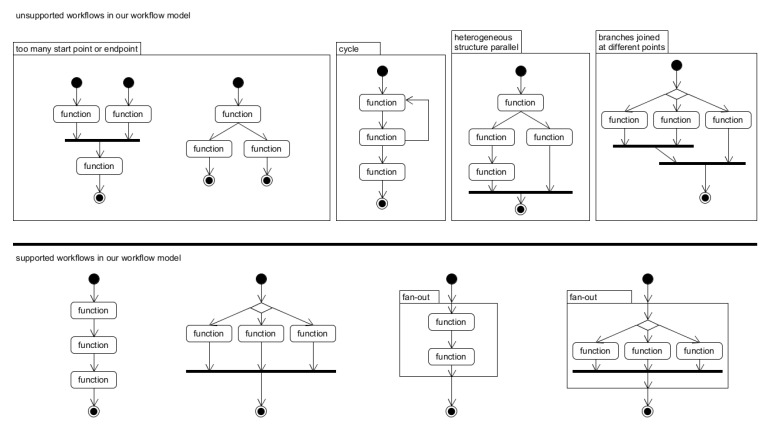
Upper five workflows are examples of unsupported workflows. The title of red boxes show why they are unsupported. Lower four workflow are supported workflows.

Figure 4 shows examples of supported as well as unsupported workflows.

### 3.2. Workflow Items

We identify three workflow items to compose a workflow.

#### 3.2.1. Function

A function is an action in serverless computing. As the implementation of the application logic, the user should provide functions in the form of source code to the serverless platform. This study assumes that all functions in each workflow are stateless (requiring all information to process as parameters and not saving any operational data) to fuse them automatically. Our assumption of stateless function is not exaggerated as data on work will be discarded after execution if the logic stores the data in persistent storage. Our method involves the execution time $T_{f}$ of each function in a workflow. This execution time includes only code running time, and not invocation latency and cold start latency. We also assume that the execution time of the function is small enough to fuse functions to make the condition suited for IoT, considering in the IoT field, workflows are often composed of functions that have short execution time [16].

#### 3.2.2. Fan-Out

The fan-out (also called dynamic parallelism or map) structure [17] is typically used to process list data. Fan-out applies the same process to each element of the given list. The process is described as a sub-workflow of the fan-out. It looks similar to iteration (for, do, while), but it is different in that the fan-out processes each element independently and runs the sub-workflow concurrently. Fan-out executes its sub-workflow (i.e., functions that will run concurrently), collects the result of each execution, and finally passes the result as a list to the next workflow item. This structure is implemented in AWS step function [18], in Azure Durable Functions [19], in OpenWhisk Composer, and in Serverless Workflow [20], which is a vendor-neutral and open-source serverless workflow composer system.

**Figure 5:**
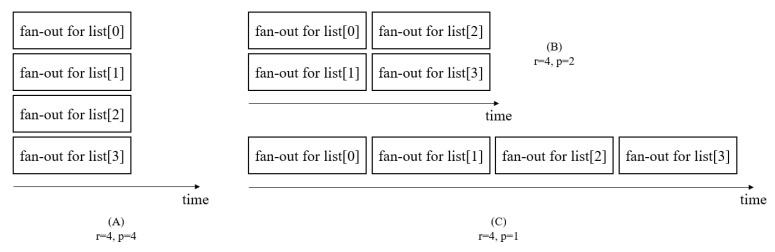
Execution pattern of the fan-out according to the maximum concurrency. The length of the input list is 4. (**A**) When maximum concurrency is the same as the number of requests, all processes can be run simultaneously. (**B**) If the maximum concurrency is half of the number of the requests, the execution time is twice that of (**A**). (**C**) If the maximum concurrency is 1, it is the same as a sequential run.

Because of an auto-scaling feature of Serverless, each execution is able to be executed in a parallel fashion. The serverless platform replicates the fan-out item and obtains as many numbers of them as asked by an input. To enable the fan-out while we consider the serverless platform’s configuration on the cloud provider, we have to make a decision on the degree of parallelism. In practice, there is a limitation for the degree of parallelism. For example, the AWS step function does not guarantee more than 40 concurrent executions of a fan-out; therefore, we need to define the maximum concurrency in our workflow fusion based on the limitation of a platform as well as the requested a concurrency (input). The number of requests for a fan-out *o* ($r_{o}$) indicates the number of elements of the list given to the fan-out *o* as iteration target. The maximum concurrency of a fan-out *o* ($p_{o}$) represents the appropriate number of executions of the fan-out *o* under the circumstance. The execution of the fan-out is bound and must be less than the number of requests. Thus, it is $p_{o}\leq r_{o}$. The execution time of the fan-out is affected by the maximum concurrency. Figure 5 shows the execution pattern according to the maximum concurrency.

#### 3.2.3. Conditional Branch

**Figure 6:**
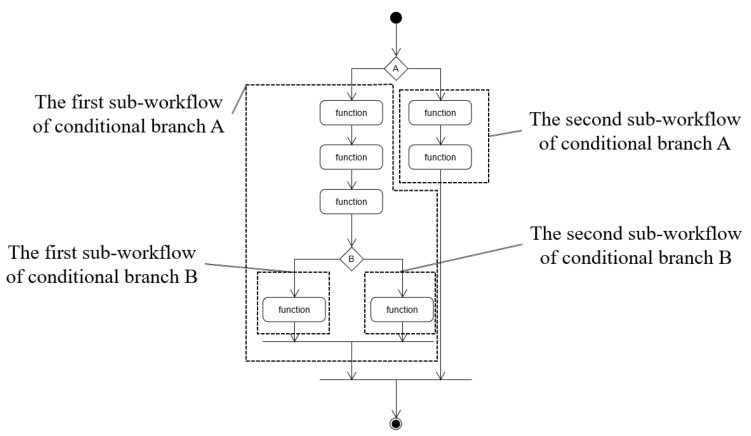
Example to show the area of each sub-flow. Sub-flow has its own sub-workflow.

At the conditional branch, the direction of the workflow is divided into at least two different paths (i.e., sub-workflow as a branch). In our workflow model, each branch must be joined at the same point. Hence, the branch is similar to an if block. This requirement is necessary to create a fused function by an if block when the condition branch is fused. The sub-workflow of a branch refers to workflow item chains that exist, from the beginning of the branch, to where branches that have split from the same conditions meet. Figure 6 presents an example that shows where a sub-workflow of a branch starts from and reaches.

Conditional branches have several possible execution paths with different execution times. If a conditional branch is fused, the fused function will show the execution time within a range according to the execution flow inside the function. One of the ways to deal with condition branches in a workflow is to determine the most likely path (MLP) as the main path, which has the highest probability to be executed among all possible paths from start to end. The processing on a workflow such as optimization or calculating response time is performed based on the MLP. Xanadu [8] uses this concept to determine what function should be loaded after a conditional branch point. Another way is that a conditional branch is dealt with as an expected value. In this way, a value such as execution time or cost of a conditional branch is calculated as an expected value. Lin et al. [21] used this method to model performance and the cost of a workflow that contained branches.

We calculated the expected value when calculating the conditional branch information. Each branch of a conditional branch has a branch probability for calculating the expected execution time of the conditional branch. Branch probability of the *i*th sub-workflow of a conditional branch cb ($pr_{cb}^{i}$) is obtained from the profiling data, for which the following should be met:

$$\sum_{i=1}^{n(cb)}pr_{cb}^{i}=1$$

where n(cb) is the number of branch in conditional branch cb.

### 3.3. Latency

In this section, we identify three types of latencies in the workflow system: cold start, invocation, and fan-out. If these latencies are ignored or included in the execution time of functions, the estimated workflow response time can be inaccurate when the workflow consists of a long chain of functions or has fused functions. Hence, our workflow response time model contains the three latencies to estimate more precisely in such cases.

- *Cold Start latency*$L_{cold}$: We define the cold start latency as a response time difference of a single function between cold start and warm start. As mentioned in the Background section, we assume that the cold start latency is the same for all functions. This latency is removed at a warm start or at the time of fusing.
- *Invocation latency*$L_{invo}$: In a workflow, there is a latency to call the following function because the serverless platform encodes the result of the previous function to general data format (usually JSON) and decodes it to call the following function. We define this latency as the difference between the end time of the previous function and the start time of the following function at the warm start mode. This latency will appear not only at the cold start but also at the warm start. However, fusing can remove it because function calls are performed directly in a thread with almost zero delays.
- *Fan-out latency*$L_{fan}$: When an execution flow reaches a fan-out, a time is needed to split the list data into each element and initiate the sub-workflow of the fan-out. We call this latency the fan-out latency and define it as the difference between the end time of the previous workflow item and the earliest start time among all instances of the sub-workflow of the fan-out. We assume that the fan-out latency is the same for all fan-outs. This latency will appear at the cold start and at the warm start but can be removed by fusion.

### 3.4. Workflow Response Time Model

In this section, we formulate the response time of a given workflow when a cold start occurs. Let G=(V,E) be the workflow represented as a DAG, where *V* is a set of workflow items (functions, fan-outs, conditional branches), and *E* is a set of call dependencies. The response time of workflow G at cold start is a recursive function:

(1) $$T\left(G=\left(V,E\right)\right)=\sum_{v\in V}\left\{\begin{array}{cc} L_{cold}+L_{invo}+T_{v}, & \mathrm{if}\;v\;\mathrm{is}\;a\;\mathrm{function}\\ L_{fan}+\frac{r_{v}}{p_{v}}T\left(G_{v}\right), & \mathrm{if}\;v\;\mathrm{is}\;a\;\mathrm{fan}\text{-}\mathrm{out}\\ \sum_{i=1}^{n(v)}pr_{v}^{i}T\left(G_{v}^{i}\right), & \mathrm{if}\;v\;\mathrm{is}\;a\;\mathrm{conditional}\;\mathrm{branch} \end{array}\right.$$

where $G_{v}$ is a sub-workflow of a fan-out *v* and $G_{v}^{i}$ is the *i*th sub-workflow of a conditional branch *v*. where $G_{v}$ is the a sub-workflow of a fan-out *v*, and $G_{v}^{i}$ is the *i*th sub-workflow of a conditional branch *v*. The response time at the warm start can be obtained by setting $L_{cold}=0$.

Hence, our goal is to reduce the workflow response time in the cold start mode by removing $L_{cold}$ using function fusion while preventing the workflow response time (*T*) from increasing.

## 4. Mitigating Cold Start Problem with Function Fusion by Considering Conditional Branch Selection and Parallelism

The fusion of two consecutive functions in a workflow prevents the cold start latency of the second function. However, when a fan-out is fused, the execution time of the fan-out increases because parallelism is also sacrificed. If the increased execution time from the loss of parallelism is longer than the removed cold start latency, the fusion will worsen the problem. The proposed scheme determines the optimal fusion strategy. To predict the total workflow time and to determine whether the function fusion is effective in reducing the total workflow execution time, it traverses the workflow, fuses two consecutive workflow items and compares the results.

### 4.1. Fusion Decision on a Function

Let $f_{a}$ and $f_{b}$ be two successive functions in a given workflow, where $f_{a}$ is followed by $f_{b}$. Using the workflow response time model defined in Equation (1), $\left(L_{cold}+L_{invo}+T_{f_{a}}\right)$ and $\left(L_{cold}+L_{invo}+T_{f_{b}}\right)$ are the response time of the two functions before the fusion, respectively. When these two functions are fused, the cold start and invocation latencies of function b are removed. Thus, our scheme will fuse any two successive functions to remove one cold start as long as the response time does not increase.

After fusing, two functions become a new function $f_{new}$, and we can replace $f_{a}$ and $f_{b}$ with $f_{new}$, whose execution time is $T_{f_{new}}=T_{f_{a}}+T_{f_{b}}$.

### 4.2. Decision for a Fusion on a Fan-Out

Let *f* be a function and *o* be a fan-out, where *f* is followed by *o*. Here, $r_{o}$ is the number of requests, $p_{o}$ is the maximum concurrency, and sub-workflow $G_{o}$ is the sub-workflow of fan-out *o*. The sub-workflow of a fan-out can be executed simultaneously with the maximum concurrency. In other words, because the execution time can overlap, the execution time of the fan-out is calculated by dividing the maximum concurrency from the sum of the response times of the sub-workflows of the fan-out and adding the fan-out latency. The response time of the workflow before fusion is expressed as follows:

(2) $$\left(L_{cold}+L_{invo}+T_{f}\right)+\left(L_{fan}+\frac{r_{o}}{p_{o}}T\left(G_{o}\right)\right)$$

If the fan-out is fused with the previous function, the fan-out latency is removed, but it loses its parallelism. Hence, Equation (2) is changed to

(3) $$L_{cold}+L_{invo}+\left(T_{f}+r_{o}T_{G_{o}^{fused}}\right)$$

where $T_{G_{o}^{fused}}$ is the execution time of a fused function that fuses all functions in the sub-workflow $G_{o}$ of the fan-out *o*. When a fan-out is fused, the sub-workflow of the fan-out should also be fused. As noted, a fan-out will be fused if the gain of removing latency is greater than the loss of increased execution time of the sub-workflow of the fan-out in our scheme. Based on Equation (4), the proposed scheme will decide on fan-out fusion.

TheEquation(2)>TheEquation(3)

(4) $$L_{fan}+\frac{r_{o}}{p_{o}}T\left(G_{o}\right)>r_{o}T_{G_{o}^{fused}}$$

If Equation (4) is true, then the fan-out is fused with the previous function, and function *f* and fan-out *o* are replaced by a new function $f_{new}$ in workflow G. The execution time of the new function is $T_{new}=T_{f}+r_{o}\sum_{f^{\prime }\in V_{G_{o}}}T_{f^{\prime }}$ because $T_{G_{o}^{fused}}=\sum_{f^{\prime }\in V_{G_{o}}}T_{f^{\prime }}$.

If a fan-out is a single-depth fan-out (that is, the sub-workflow of the fan-out contains only functions, not a fan-out or conditional branch.), the response time of the sub-workflow of fan-out $T(G_{o})$ can be represented as

$$T\left(G_{o}=\left(V_{G_{o}},E_{G_{o}}\right)\right)=\sum_{f\in V_{G_{o}}}\left(L_{cold}+L_{invo}+T_{f}\right).$$

Hence, Equation (4) for a single-depth fan-out is

$$L_{fan}+\frac{r_{o}}{p_{o}}\sum_{f^{\prime }\in V_{G_{o}}}\left(L_{invo}+L_{cold}\right)>\frac{r_{o}\left(p_{o}-1\right)}{p_{o}}\sum_{f^{\prime }\in V_{G_{o}}}T_{f^{\prime }}.$$

If the above is true, fuse the single-depth fan-out is fused.

If the sub-workflow of the fan-out (upper fan-out) contains other fan-outs (inner fan-out) or conditional branches (inner conditional branch), our scheme first tries to fuse these inner fan-outs and conditional branches and replace them with fused functions if possible. If all inner fan-outs and conditional branches in the sub-workflow of the upper fan-out can be fused, the sub-workflow of the upper fan-out becomes a linear function chain, that is, the upper fan-out is now a single-depth fan-out. Conversely, if any inner fan-out or conditional branch in the sub-workflow of the upper fan-out cannot be fused, the upper fan-out will not be fused. If the upper fan-out is fused, inner fan-outs and conditional branches also should be fused with the upper fan-out. Hence, the upper fan-out is not fused because an inner fan-out or conditional branch in the sub-workflow of the upper fan-out has been determined as one that should not be fused. In our scheme, this fusion decision process will be applied recursively.

### 4.3. Fusion Decision on a Conditional Branch

Let *f* be a function and cb a conditional branch, where *f* is followed by cb. The conditional branch cb has information about sub-workflows of its branches and the branch probabilities (i.e., probabilities for each branching direction). The sub-workflow of the *i*th branch of the conditional branch cb is denoted as $G_{cb}^{i}$, and the branch probability of the *i*th branch is denoted as $pr_{cb}^{i}$.

Similar to the fan-out decision process, our scheme first attempts to fuse the sub-workflow of each branch. If a sub-workflow of a branch has a fan-out or conditional branch, this process is applied recursively. Subsequently, if all sub-workflows of the conditional branch can be fused, the conditional branch is fused with the previous function because the sub-workflows of the condition branch are the same as the serial function chain because only one is executed at a time point. After fusion, the scheme replaces the conditional branch and its sub-workflows with a new function in workflow *G*. With the replacement (i.e., after branch fusion), the execution time of the new function is determined by the expected time formula:

$$T_{new}=T_{f}+\sum_{i=1}^{n(cb)}pr_{cb}^{i}T_{{\left(G_{cb}^{i}\right)}^{fused}}$$

where $T_{{\left(G_{cb}^{i}\right)}^{fused}}$ is the execution time of a fused function that is from *i*th sub-workflow of the conditional branch cb.

### 4.4. The Overall Process

Algorithm 1 shows the overall process of our optimal fusion strategy. The algorithm requires workflow information as an input and the primary return value is a set of fusion sets. A fusion set is a set of functions that has to be fused into a single function. This algorithm starts from the first workflow item of the given workflow and determines fusion of the current workflow item with the previous item. After a decision on the current item is reached, it moves on to the next workflow item until it reaches the last workflow item

The Boolean return value “fusible” is used for the recursive call. The false value of fusible represents the fact that there is a non-fusible workflow item in a given workflow. Thus, the upper workflow item will not be fused. If there is a non-fusible function in the middle, the algorithm makes a new fusion set for the next functions.

**Algorithm 1:** RAOFS: The Recursive Algorithm for Optimal Fusion Strategy

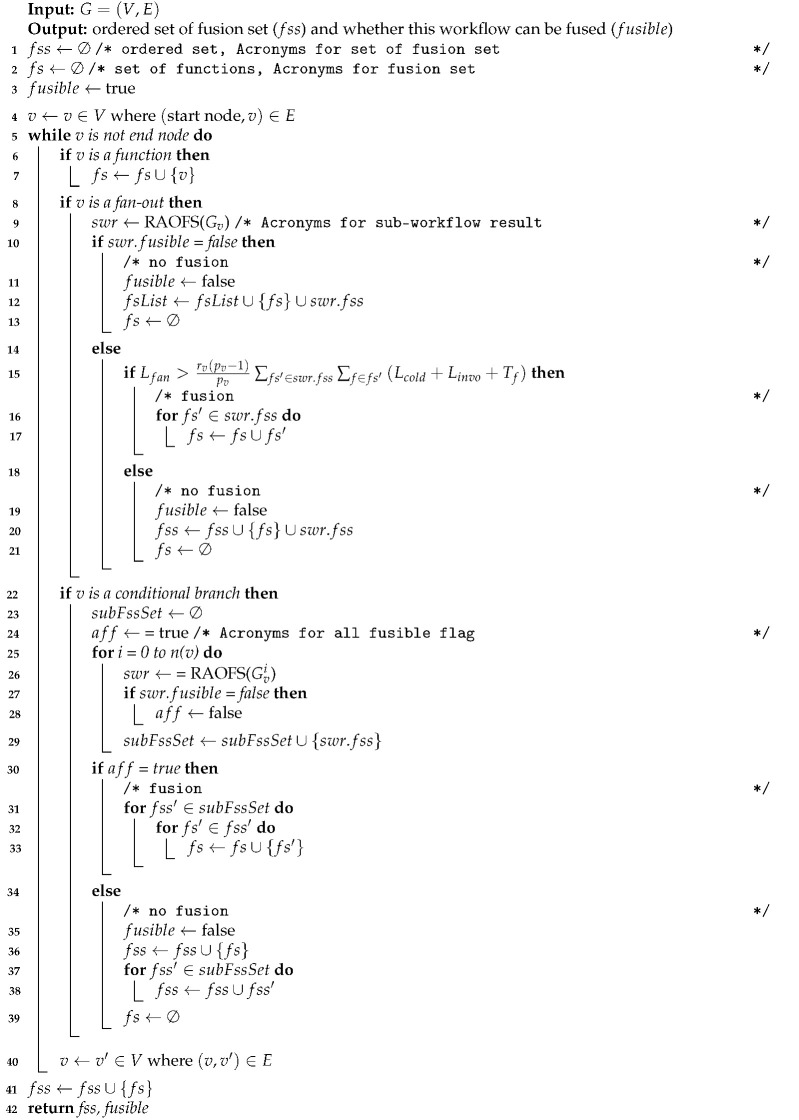



### 4.5. Writing a Fused Function

In this section, we illustrate the coding process of the fused function. We use the process in our evaluation prototype for fusion automation, for which we made several assumptions in addition to our overall workflow assumption.

- All source functions are stateless. They do not store any state data for future use.
- The input and output of a source function are a single object that may contain multiple properties.
- If a source function contains an asynchronous call, it will be blocked until resolved.

In our scheme, a mediator function is required to fuse two functions into a single fused function. A mediator function coordinates source functions and passes input and output data between source functions. For this, the mediator function needs to load source functions to call. One of the ways to load a module is to use the module system provided by the programming language. Many programming languages support module systems for code reusability, for example, “require” keyword in commonJS and “import” keyword in python. In the module system, one module can use the other modules without invading their namespace or scope. A function can be a module, and thus, the mediator call sources functions as modules. The module system can separate each source function into its own scope. Hence, no collision occurs even if the two functions depend on different versions of the same module.

**Figure 7:**
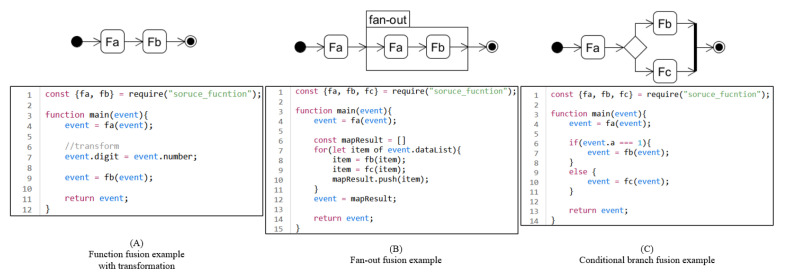
Example to show how to write fused function in JavaScript. (**A**) shows the case of two functions. (**B**) shows the case of a fan-out. (**C**) shows the case of a conditional branch.

The mediator function receives the event input from the workflow as its function parameter. Then it passes the event to the first source function and delivers the result to the second source function. The mediator function returns the result of the last source function as its result. If two successive functions have different message structures, the mediator function transforms the message format for functions. Figure 7A depicts a fused function example written in JavaScript. In this example, two function fa and fb are in a line. The function named main is the fused function. Function main gives its input to the first source function fa. At line number 7, the property name of the out of fa is changed from “number” to “digit” because the next function fb requires “digit”. For the last, the function main returns the output of the function fb.

When a mediator function includes a fan-out structure, the fan-out is transformed to the for-each block provided by its programming language. It iterates the sub-workflow of the fan-out and collects each result of the sub-workflow as an array. For example, JavaScript provides for-of keyword for for-each patten. Figure 7B shows the fusion example of a fan-out. In this example for-of block of JavaScript is used to handle the iteration. Before the iteration starts, an array is created to save the result of each iteration. Each iteration saves the result at the last (line 10). Finally, the array becomes the result of the fan-out (line 12).

When a mediator function includes a conditional branch structure, the conditional branch is transformed to an if block provided by its programming language. Only one sub-workflow is executed according to the conditions, and the results are delivered to the following function. Figure 7C shows a fused function example written in JavaScript.

**Figure 8:**
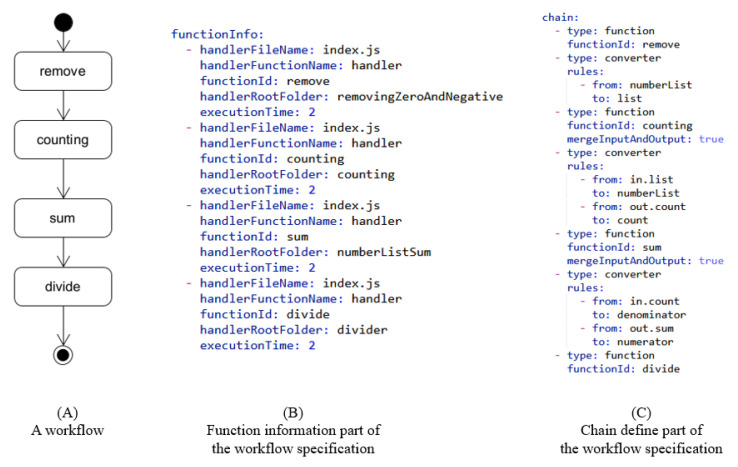
Example to show the workflow specification written by YAML. This workflow specification contains message transformation steps.

We implemented the fusion automation process based on this scheme using TypeScript on Node.js v14.0.0. The fusion automation can support only AWS Lambda format, but it can be easily can easily be extended to other serverless platforms. We define a format based on JSON/YAML to describe a workflow. Figure 8 is an example of workflow specification. The source codes of the algorithms and a document of JSON schema for the workflow specification can be used in our Github repository (https://github.com/henry174Ajou/AWS-Lambda-Fusion-Automation (accessed: 15 December 2021)).

## 5. Experiment

### 5.1. Experimental Setting

We used AWS Lambda for the serverless computing platform and AWS step function for a workflow system to evaluate our scheme. We configure AWS Lambda as a function with the following common properties:

- Memory: 128 MB.
- Region: ap-northeast-2.
- Runtime: Node.js 14.x.

We implemented our scheme for optimal function fusion strategy algorithm (Algorithm 1) on Node.js v14.0.0 using TypeScript. Unfortunately, there is no representative workflow for performance evaluation in serverless computing to the best of our knowledge, because workflows in serverless computing is a relatively fresh research topic. Instead, we prepared three example workflows for verification and two real-world scenario workflows. The source functions and the work flow structure can be found in our Github repository (https://github.com/henry174Ajou/AWS-Lambda-Fusion-Automation (accessed: 15 December 2021)).

### 5.2. Example Workflows

**Figure 9:**
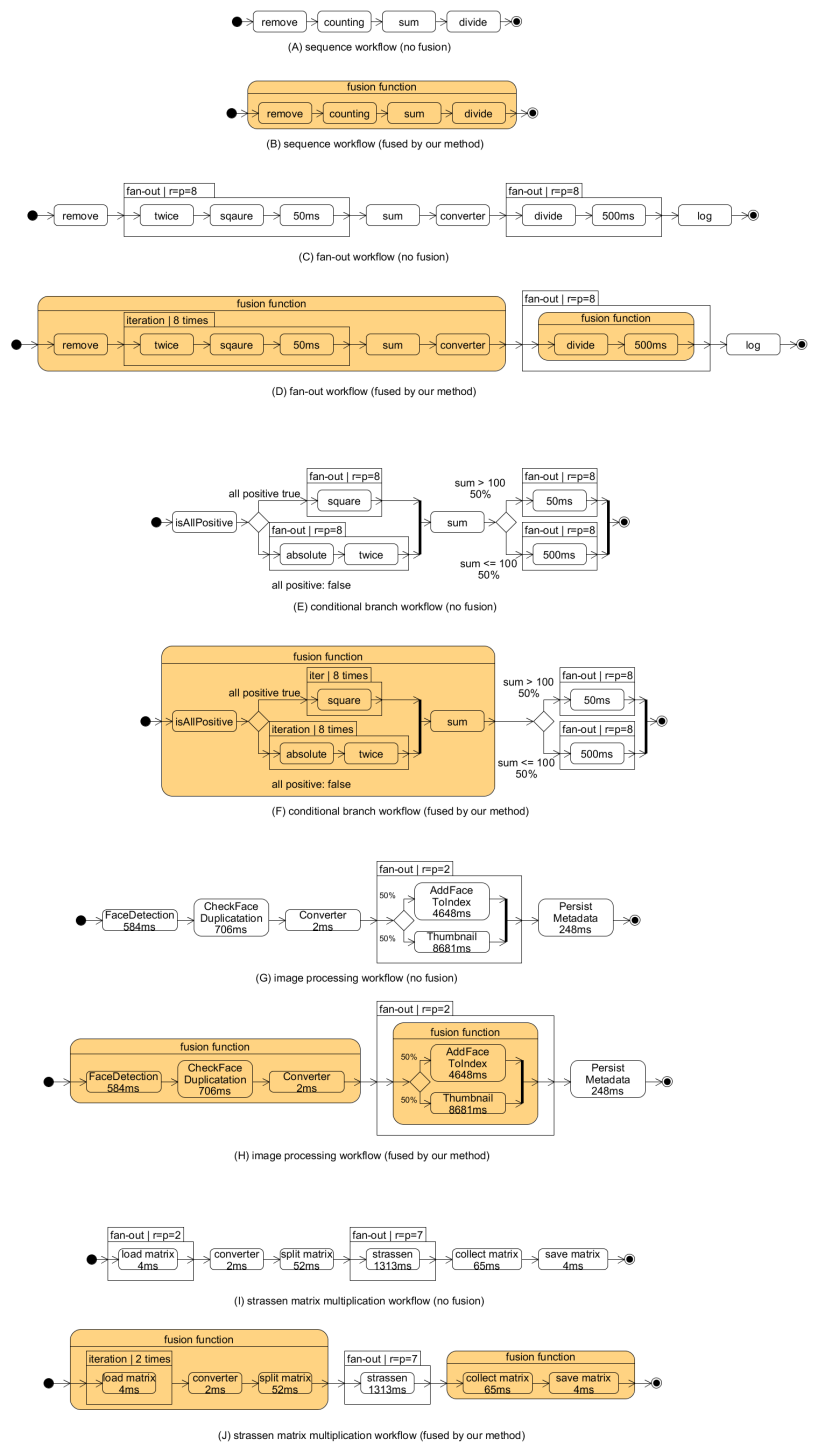
Three workflows to show that different decisions take place in some cases and two workflows that have a purpose for real-world scenario. (**A**,**C**,**E**,**G**,**I**) are original workflows that are not fused, (**B**,**D**,**F**,**H**,**J**) are workflows fused by our method.

We prepare three example workflows to verify that our model and algorithm makes different fusion decisions on cases. Figure 9A–F show the example workflows and fusion selection of our proposed scheme. The execution time of functions whose execution time is not specified is 2 ms.

Sequence workflow of Figure 9A is prepared to verify the effectiveness of our proposed scheme for sequence of function executions. Sequence workflow receives a number array as an input and returns the average value of the positive values in the input array except for negative values. Fan-out workflow (Figure 9C) receives a number array and returns the normalized number array except for the negative values. We prepare this workflow to verify the effectiveness of our proposed scheme for repetitive function executions. With the fan-out workflow, we evaluate the benefits of concurrent execution of functions such as ‘for clause’ in function fusion. We design the workflow to have both (1) the part where function fusion does not do any harm (i.e., no increased response time) and (2) the part where function fusion does harm (i.e., no increased response time) to the performance. As represented in Figure 9E, the last example workflow receives a number array. If all elements are positive, we square each element; otherwise, we multiply two to the absolute of each element. If the sum of the array is larger than 100 after the processing of each element, each element is processed by the function whose execution time is 50 ms; otherwise, each element is processed by the function whose execution time is 500 ms. Conditional branch workflow is prepared to verify the effectiveness and correctness of our proposed scheme for conditional branch such as ’if clause’ in function fusion. We design the conditional branch workflow to have both (1) the fused branch without the increase in response time, and (2) the fused branch with the increase in response time. The fusion selection of our method is depicted in Figure 9B,D,F).

**Figure 10:**
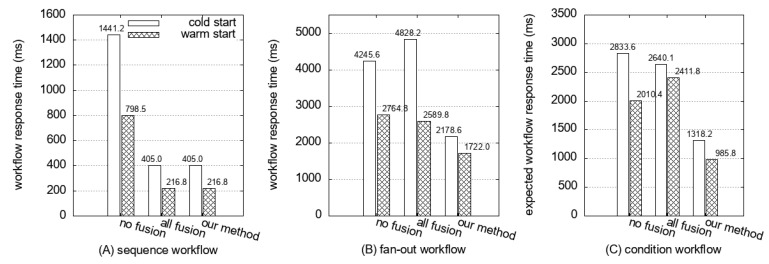
Workflow response time on the cold start and the warm start of the original workflow, all functions fused workflow, and workflow fused by our method (in example workflows).

Figure 10 illustrates the experimental results of our example workflows; “no-fusion” is the original workflows, “all-fusion” is the result when all functions of the workflow are fused into a single function, and “our method” is the result when functions are fused according to the optimal fusion strategy of our proposed scheme. We yield five cold start requests and five warm start requests to check the cold start latency. AWS Lambda reclaims the allocated resources of functions unless there is any request in 10 min (as discussed in Section 2.2). For our experiment, we invoked the workflow with a 15-min interval, which is the announced 10 min intervals plus additional 5 min for safety, to make sure that a cold start will occur. Additionally, we checked the log provided by AWS Lambda to make sure whether the cold start occurred or not after invocation.

All three experimental results of the example workflow show a similar tendency. Using the proposed method, we can reduce the overall workflow response time with reduced cold start latency compared with the all-fusion method and the original workflows. Although the all-fusion method showed a shorter cold start latency than our scheme in fan-out workflow and condition workflow, the workflow response time of all-fusion is increased due to the loss of parallelism. Our scheme also reduces the response time on the warm start because the invocation delay is removed for the fused function.

### 5.3. Real-World Workflow

#### 5.3.1. Face Detection

Wild Rydes image processing workflow [22] detects a face from a given image and checks that this person is already registered. This workflow is built for a ride-sharing app for the drivers to identify the rider during pickup as well as preventing a user to create multiple accounts for promotions.

This workflow initially has five functions, with two of them running in parallel. We modify the parallel run as a fan-out structure and add one mediator function transforming a message format to meet our workflow model. Figure 9G depicts the workflow used for the test.

**Figure 11:**
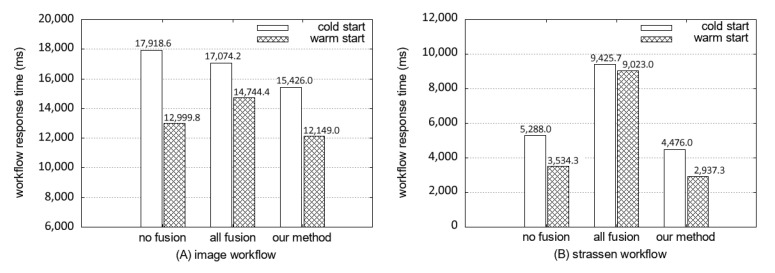
Workflow response time on the cold start and the warm start of the original workflow, all functions fused workflow, and the workflow fused by our method (in real-word workflows).

Figure 11 illustrates a similar result with the test using example workflows. The workflow based on our fusion decision shows a shorter response time of the workflow than the no-fusion and the all-fusion.

#### 5.3.2. Matrix Multiplication

The workflow of Figure 9I represents the Strassen algorithm of multiplication of two matrices. The original workflow for implementing the Strassen algorithm was designed by Werner et al. [23] to exploit the auto-scaling feature for matrix multiplication speed-up. The workflow receives the names of two input matrices. Then, matrix data are loaded from a storage. The two matrices are split into sub-matrices for the Strassen process in the function named “split matrix”. The Strassen function calculates seven intermediate terms in Strassen with the calculation process running in parallel. Finally, the collection function collects all intermediate results and generates the final multiplication result. The last of the workflow is for saving the result.

**Figure 12:**
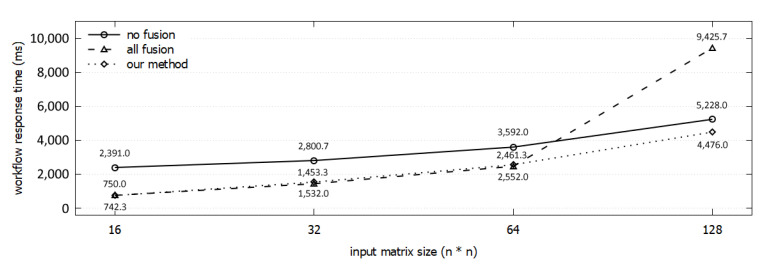
Change of the workflow response time of three methods by input matrix size.

The workflow response times of three fusion decisions are presented in Figure 11B for the size of an input matrix is 128 × 128. For all-fusion, the Strassen algorithm cannot be implemented in parallel. Consequently, it shows the largest response time among the three. In contrast, the fused workflow (Figure 9J) from our scheme shows the shortest response time because of it runs the Strassen function in parallel. We conducted experiments comparing our method with the original workflow and all-fusion workflow by increasing the matrix size. When the matrix size is smaller than 64, our scheme shows the same result as all-fusion. Figure 12 shows that the response time of the all-fusion workflow is rapidly increased because of sequential run of Strassen. In contrast, the results of our scheme follow the rate of increasing with the original workflow, but with shorter response times.

**Figure 13:**
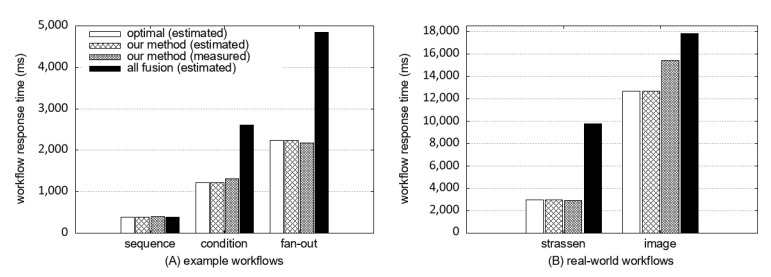
Comparing the estimated response time of our model with measured response time (ground truth) of each workflow in this paper.

We measured the accuracy of our response time model by the time difference between a predicted response time of our model and an actual workflow response time measured on AWS. We also measured the accuracy of our proposed scheme by the time difference between a predicted response time of our model and a predicted response time of the optimal solution obtained by brute force. Figure 13A shows the comparison results of the three example workflows, and Figure 13B shows the result on the two real-world workflows. The “optimal (estimated)” label represents the estimated response time from the brute force. The “our method (estimated)” is the estimated response time of the workflow obtained using our method. The “our method (measured)” is the measured response time of the workflow of our method. The “all-fusion (estimated)” label is the estimated response time of the workflow in which all functions are fused into a single function. We can see that our proposed scheme can find an almost optimal fusion strategy, and our response time model can estimate the response time after fusion.

## 6. Related Works

This section introduces studies related to reducing the cold start latency or the function fusion in a workflow.

### 6.1. Workflow in Grid/Cloud and Serverless Computing

The concept of processing tasks as a workflow has been around for a while. Even though workflows are an efficient tool to organize and coordinate various tasks; however, it is hard to port the workflow and its execution environment to others because each task in the workflow has dependencies. To address the issue, various workflow systems and related schemes have been proposed for the cloud computing environment [24] and grid computing environment [25] to enhance the portability, i.e., to make it easy to re-implement the environment by capturing dependencies and virtualizing the underlying system (in cloud computing) [24] or sharing hardware configured for the required environment with other organizations (in grid computing) [25]. However, there is room to improve for those suggestions. For example, using the proposed approach [24] may increase the system complexity because of its component-reusing mechanism. Even though those proposals achieved a certain level of portability, those suggestions can be improved in reusability. Since reusability is one of the essential properties of workflow, researchers continue to study to provide efficient software reuse architectures and systems in serverless computing. In serverless computing, the workflow consists of functions, which perform a unit task and have their own dependencies by isolation. Baldini et al. [10] proposed a workflow system in serverless computing that allows users to build a software by just assembling existing software blocks. Since it defines the functions as black-box blocks, the proposed scheme is more robust to the reusability problem.

### 6.2. Mitigating Cold Start by Function Preloading

Xanadu [8] is an optimization algorithm that reduces the total cold start latency of a workflow and waste of resources due to preloading. The main idea behind Xanadu is that if it can be known which function will be called next, the following function can start warm by deploying a new instance of the function in advance; however, if a serverless platform preloads functions too early, the resources are wasted due to over-provisioning. Hence, Xanadu used a just-in-time preloading approach to minimize the time a preloaded function waits for the actual request to arrive. In addition, Xanadu defined the most-likely-path (MLP) concept in a workflow to handle conditional branches. The MLP refers to the path with the highest probability of execution among several paths (function chain) with starting to end points of a given workflow. Xanadu preloads functions along with the MLP. When the execution flow misses the MLP, Xanadu calculates the new MLP based on the current flow, revokes incorrect preloaded functions, and preloads the following function through the new MLP; however, Xanadu requires modifying a serverless platform. It is not easy to use this approach if the user does not have the right to modify a serverless platform, especially a commercial serverless platform. In contrast, our proposed technique has more applicability than Xanadu because fusion can be implemented at the client-side, and a fused function is no different from the normal function. In other words, fusion is an independent process from serverless platforms.

### 6.3. Reducing the Cost of Serverless Computing by Function Fusion

Costless [11] is an optimization algorithm for saving operation costs using a commercial serverless platform with IoT devices. Workflow composer systems of commonly used commercial serverless platforms charge the fee of not only the function execution time but also the number of function invocations. The main idea in Costless is that if functions are fused into one single function, then the number function invocation is also reduced to just one. In addition, Costless used on-premise IoT devices to run functions to save function execution fees. Costless determines which function remains on the cloud, which function moves to IoT devices, and whether to fuse the functions listed consecutively in the cloud from a cost perspective and constraint with a maximum response time of the workflow. Costless is an optimization algorithm to save operation costs using a commercial serverless platform with IoT devices.

Each decision is represented as a label of a vertex in a directed acyclic graph. Each path from start to end represents a possible solution, and the total weighted sum of the path means the total cost and response time. Using these modeling approaches, Costless could convert the optimization problem to the constrained shortest path problem, which is an NP-hard problem but has a fast heuristic with a proven approximation ratio.

However, Costless can handle only a linear function chain. If there is a branch in a given workflow, the user should linearize the workflow into one possible linear chain to use Costless. If a workflow contains parallel steps, the users should abandon parallelism. However, our proposed method can consider branches and parallel steps. Our method can exploit parallelism by comparing the case of parallel and linearizing the parallel step.

### 6.4. Workflows Management and Optimizations for Serverless Computing

Many optimization methods for complex workflows have been proposed in recent studies. In [21], authors define common patterns for workflows (i.e., self-loop, cycle, parallel, branch) as probabilistic direct acyclic graphs to model various types of workflows. Their method optimizes the performance of the cost of a workflow using a greedy heuristic that adjusts memory configuration based on the critical path. In contrast, serverless deployment of deep learning models has been widely studied because its complex workflow makes it difficult to be used in a serverless environment. In Gillis [26], parallel workflows in deep learning inferences are simplified by a linear graph model. Using the linear graph model, they proposed a dynamic-programming-based method and a reinforcement-learning-based model to optimize the response time and cost, respectively.

## 7. Conclusions

We presented the impact of the cold start problem on a serverless workflow. Our study is focused on a serverless workflow with a parallel execution structure, where the response time may increase when the workflow is fused. The goal of our proposed scheme is to reduce the workflow response time by the function fusion while considering branches and parallelism. To achieve this, we identified three workflow items (i.e., function, fan-out, and conditional branch) and three latencies (i.e., cold start latency, invocation latency, and fan-out latency). Based on this definition, we modeled the problem to estimate the response time of a workflow with the conditions for whether to fuse or not. We showed that fusion can reduce the response time for both the warm start and cold start of the workflow. As shown in the experimental results, for a workflow of nine functions, our scheme reduces the response time at the cold start by almost half compared with the original workflow; however, when all nine functions were fused, the response time increased by 14%. Our proposed scheme can be used to find an almost optimal strategy or a high-quality solution. In this paper, we evaluated our method on the public cloud (AWS Lambda). We plan to extend our study and to use serverless platforms such as OpenWhisk [27] and OpenFaaS [28], which will allows us to consider issues such as data locality and provisioning. We expect that this further study makes the fusion decision more sophisticated.

As already suggested in Baldini et al. [10], function fusion is one of the possible solutions for the double-spending problem [29] in which the cost for a scheduler function that is used to compose a given workflow is charged in addition to the cost for source functions. Our proposed method is based on function fusion, so it may be used to remove the scheduler function to reduce doubled spent cost. However, billing models vary from provider to provider, and they may change over time even in the same provider (i.e., the cost model is difficult to generalize). We are concerned that the cost model may be subordinate to a particular vendor.

We also provided schemes for function fusion automation and its implementation for the AWS Lambda. The scheme is only supported for AWS Lambda; however, it can be easily extended to other serverless platforms. In future work, we will extend our scheme by adding more fusibility with workflow reconfiguration such as topology change.

## Figures and Tables

**Table 1:** Symbols used in this paper.

Symbol	Meaning
$T_{f}$	execution time of function *f*
G=(V,E)	a workflow represented in DAG
T(G)	response time of workflow *G*
$r_{o}$	the number of requests to a fan-out *o*
$p_{o}$	the maximum concurrency of a fan-out *o*
$G_{o}$	the sub-workflow of a fan-out *o*
$pr_{cb}^{i}$	the branch probability of the *i*th branch of a conditional branch cb
$G_{cb}^{i}$	the *i*th sub-workflow of a conditional branch cb
$L_{cold}$	the cold start latency
$L_{invo}$	the function invocation delay of the workflow system
$L_{fan}$	the fan-out delay to initiate a fan-out of the workflow system
n(cb)	the number of sub-workflow of a conditional branch cb

## Data Availability

Datasets derived from public resources and made available with the article. All Source functions and workflow specifications used in this paper are available on the following Github repository. https://github.com/henry174Ajou/AWS-Lambda-Fusion-Automation (accessed: 15 December 2021).
